# Gaining or Losing Team Ball Possession: The Dynamics of Momentum Perception and Strategic Choice in Football Coaches

**DOI:** 10.3389/fpsyg.2019.01019

**Published:** 2019-05-28

**Authors:** Walid Briki, Bachir Zoudji

**Affiliations:** ^1^ Sport Science Program, College of Arts and Sciences, Qatar University, Doha, Qatar; ^2^ EA 2445, DeVisu, Laboratoire en Design Visuel et Urbain, Université Polytechnique Hautes-de-France, Valenciennes, France

**Keywords:** dynamics, momentum, strategy, coaches, football

## Abstract

Grounded in the dynamical systems approach, the present research examined the influence of team ball possession (TBP) in soccer on coaches’ perceived psychological momentum (PM) and strategic choice (i.e., game-based “stick” vs. “switch” choices) during a simulated match. Experienced soccer coaches imagined being the coach of the team involved in a highly important match that was displayed on a wall in a lecture hall. The match scenario was manipulated so that the coach was exposed to either a positive momentum sequence (i.e., ascending scenario of TBP) or a negative momentum sequence (i.e., descending scenario of TBP). Results revealed that positive (or negative) momentum sequence increased (or decreased) perceived PM and increased stick (or switch) choices. Perceived PM globally evolved linearly, while strategic choice displayed a dynamical pattern of “critical boundary” (thus showing a nonlinear change). Nonetheless, both variables displayed asymmetrical effects, in the sense that: (1) the strength of positive PM appeared to be easier to decrease than to increase; and (2) the greater the positive PM (or the negative PM), the lesser (or the greater) the coaches’ tendency to make a change in the organization of their teams. This investigation evidences that TBP can powerfully influence coaches’ perceptions and strategic decisions, and that coaches are more likely to be sensitive to negative events than to equivalent positive events.

## Introduction

Psychological momentum (PM) refers to a “psychological force in which several factors or qualities converge in a synergistic way to enable one to perform at a level not ordinarily possible” (Iso-Ahola and Dotson, 2014, p. 20). PM is thought to take place in a large variety of contexts, such as economics, politics, and sport (Adler, 1981). In sport psychology, studies characterized PM as a force that is either positive or negative and that can be experienced from the standpoint of either *actors* (e.g., Jones and Harwood, 2008; Briki et al., 2013) or *observers* (e.g., Briki et al., 2014a, 2016). Positive PM refers to periods of the game where people experience that everything is going smoothly, while negative PM corresponds to opposite situations where people perceive that everything is going wrong. Studies conducted on PM in sport mostly investigated either athletes (as actors) or supporters (as observers), and thus much less concerned coaches (Moesch and Apitzsch, 2012). Yet, coaches occupy a position that combines both the role of observer (when they watch and follow the actions of their athletes during the ongoing game) and that of actor (when they make decisions and seek to adjust or optimize athletes’ actions during the ongoing performance). Therefore, the present study aimed at examining the experience of PM from the viewpoint of coaches.

Several PM models and empirical investigations attempted to identify factors that could trigger PM over time and link PM to affective, cognitive, and behavioral components (e.g., Adler, 1981; Vallerand et al., 1988; Taylor and Demick, 1994; Markman and Guenther, 2007; Gernigon et al., 2010; Briki et al., 2013, 2016; Den Hartigh et al., 2014). Three types of PM triggers have been identified, such as psychological (e.g., emotional or cognitive states, energy), environmental (e.g., score, critical events or actions, climatic conditions, referees’ decisions), and social (e.g., teammates’ behavior, coaches’ instructions) determinants (e.g., Adler, 1981; Taylor and Demick, 1994). Despite this, numerous studies used *outcome-related patterns* (e.g., score, score-gaps, time-gaps) to manipulate positive PM and/or negative PM (e.g., Vallerand et al., 1988; Markman and Guenther, 2007; Gernigon et al., 2010; Briki et al., 2013, 2014a,b, 2016). However, although outcomes can powerfully influence the perceived PM of athletes and supporters, they hardly reflect the complex reality of games, which encompass series of actions that can be assessed through a wide range of behavioral parameters. For example, in some collective sports like soccer, quality of collective tactics, team technical mastery, ball possession, etc., are of considerable importance and can powerfully determine the ultimate outcomes (Travassos et al., 2013). Therefore, a major goal of the present research was to examine whether team ball possession (TBP), considered as a critical behavioral variable of performance in soccer (Jones et al., 2004; Lago and Martín, 2007; Oberstone, 2009; Lago-Ballesteros and Lago-Peñas, 2010), could influence the PM experience of coaches and their decision-making.

### Psychological Momentum and Its Properties

Drawing a loose analogy to Newtonian physics, Adler (1981) and Markman and Guenther (2007) conceptualized PM through a psychological velocity (v) × psychological mass (m) formulation (v *×* m) attempting to capture the phenomenological experience of PM. “Psychological velocity” refers to the perceived speed of movement toward the desired or undesired goal, while “psychological mass” corresponds to the perceived importance of the given situation. According to v *×* m formulation, the combination of psychological velocity with psychological mass gives rise to the experience of PM. Markman and Guenther (2007) assume that people possess naïve beliefs or implicit theories, which refer to knowledge that people use to make their surrounding world meaningful (e.g., Heider, 1958), can affect psychological velocity and psychological mass that underlie the experience of PM. In a study designed to examine the effect of environment on PM, Briki et al. (2016) found that sport supporters involved in an uncomfortable climate condition (i.e., hot-wet environmental climate) reported higher perceived PM than did those involved in a comfortable climate condition (i.e., neutral environmental climate). The authors attributed such a result to the influence of a naïve belief acting as an augmenting inference based on the imagined discomfort that the supported athlete was supposedly experiencing during the competition (e.g., “He’s leading the race despite the very hot weather!”). A more basic naïve belief that is supposed to underlie the experience of PM is that PM affects performance, which may lead lay perceivers to develop specific perceptions and judgments about the actual and expected outcomes (e.g., Markman and Guenther, 2007; Briki et al., 2013).

Based on the view that the development of perceptions and judgments is often evolving, changing, and dynamical (Nowak and Vallacher, 1998), we borrowed the approach of dynamical systems (Kelso, 1995; Nowak and Vallacher, 1998) in order to investigate and explore the phenomenon of PM. According to Gernigon et al. (2010), PM can be construed as a dynamical system, defined as a set of interrelated determinants changing over time that may display properties of *nonlinearity* and *history-dependence*. PM nonlinearity refers to the proneness of PM to display qualitative changes over time that reflects a disproportionate relationship between causes and consequences (Nowak and Vallacher, 1998). As for PM history-dependence, it refers to how a momentary state of PM is dependent on either its previous states (reflected through change resistance) or its anticipated states (reflected through anticipated escape of the momentary state of PM toward another state) (Nowak and Vallacher, 1998). In order to test the properties of nonlinearity and history-dependence, Haken et al. (1985) recommended the use of the synergetic approach, which refers to an interdisciplinary field of research dealing with the formation of spontaneous patterns. This approach indicates that the investigation of such properties requires the linear and gradual manipulation of a *control parameter* (i.e., a variable that can lead the investigated phenomenon through its different states). For example, several studies conducted on PM induced PM by using either ascending or descending scenarios of score-gap or time-gap between the opponents (e.g., Gernigon et al., 2010; Den Hartigh et al., 2014; Briki et al., 2016). In these studies, the control parameter corresponded to the score-gap or time-gap, depending upon the studies. Furthermore, studies seeking to identify the keys to success in soccer showed that highly successful teams displayed higher levels of ball possession, long passes, and shots at goals, as compared to less successful teams (Travassos et al., 2013). Hence, in line with the synergetic approach, the amounts of these events could be considered as potential control parameters of success and, thus, of PM in soccer.

**Figure 1:**
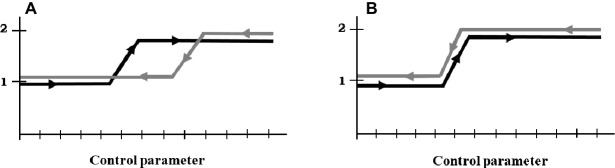
Schematic representation of the **(A)** negative hysteresis and **(B)** critical boundary. This figure is adapted from that of Briki and Markman (2018). The ascending scenario in black (or the descending scenario in gray) refers to a scenario in which the control parameter changes in an ascending (or descending) way, i.e., from the lower (or higher) values to the higher (or lower) values. On the *y*-axis, 1 and 2 correspond to possible states that characterize a given system.

Recent studies have investigated the properties of nonlinearity and history-dependence while participants were either supporting their preferred athlete (Briki et al., 2014a, 2016) or performing physical efforts (Briki et al., 2013) within important competitions. These studies revealed different results depending upon the type of task involvement. Specifically, the authors showed, in supporters, an early shift of perceived PM in response to ascending and descending time-gap scenarios, giving rise to a “negative hysteresis” pattern (Kelso, 1995; see Figure 1A). The pattern of negative hysteresis indicated that PM perceptions shifted early both when the supported athlete appeared to be losing the lead while still being ahead of the other athlete (at the beginning of the descending momentum sequence), and when approaching the lead while still being behind the other athlete (at the beginning of the ascending momentum sequence). The authors suggested that such a dynamical pattern would reflect how observers experience PM while mentally simulating a possible future of winning or losing on the basis of initial events (e.g., Markman et al., 2009). In addition, the authors reported that the early shift was more rapid or abrupt in the ascending sequence compared to the descending sequence, suggesting a proclivity of supporters toward *self-serving bias* (e.g., Babad, 1987). Briki et al. (2013) showed, by contrast, that athletes’ PM revealed a “critical boundary” pattern (Kelso, 1995; see Figure 1B) in that the change in perceived PM in both ascending and descending conditions occurred at the same performance event. In addition, the shift was more abrupt in the descending than in the ascending sequence, which the authors suggested showed that actors were more subject to *loss aversion* (Kahneman and Tversky, 1979).

### Psychological Momentum and Strategic Choice

Although people’s lay theories can affect PM and subsequent judgments, the literature of PM reveals a lack of studies seeking to examine how PM may influence judgment and reasoning, such as strategic choice (i.e., “stick” vs. “switch” choices) (Guenther and Kokotajlo, 2017). To date, only the study of Guenther and Kokotajlo (2017) has investigated whether and how PM could affect actors’ choices, and this study took place in the context of finance. The results of their study showed that positive PM increased switch choices through increased sense of self-confidence. This result echoes Attali’s (2013) archival analysis showing that NBA players attempted unusual shots after a successful shot (i.e., switch choice) compared to after missing a shot. Nonetheless, and interestingly, Attali (2013) observed that coaches were more likely to make the decision not to remove the player who was successful (i.e., stick choice). Finally, if we apply Attali’s findings to the perspective of PM, one might expect that a coach seeing that his/her team is moving from behind to lead (i.e., involved in a positive momentum sequence) would tend to opt for stick choices, whereas a coach seeing that his/her team is losing the lead and getting behind (i.e., involved in a negative momentum sequence) would tend to opt for switch choices.

### Research Overview

In addition to examining whether TBP could trigger the perceived PM of coaches, the present research sought to see whether coaches’ perceived PM might display nonlinearity and history-dependence properties. This research also aimed at examining how momentum sequences may affect coaches’ choices (in terms of either stick or switch choices in the organization of their teams). Because high percentage of ball possession was identified as a key to success in soccer (e.g., Lago and Martín, 2007; Lago-Ballesteros and Lago-Peñas, 2010), we expected coaches’ perceived PM (1) to increase in the ascending sequence of TBP percentage (i.e., positive momentum sequence), and (2) to decrease in the descending sequence of TBP (i.e., negative momentum sequence). Based on Attali’s findings on coaches’ choices, we expected coaches (1) to make fewer changes while experiencing more successes (i.e., positive momentum sequence), and (2) to make more changes while experiencing more failures (i.e., negative momentum sequence). Regarding the nonlinearity and history-dependence properties, previous studies displayed differentiated reactions according to the type of task involvement as actor vs. observer (e.g., Briki et al., 2013, 2014a). Nonetheless, because coaching athletes consists in observing, supporting, and acting on athletes’ behaviors, thus combining the roles of observer and actor, no hypothesis could be formulated on the dynamical properties of coaches’ perceived PM. In order to provide a valid and rigorous test, we adopted two essential methodological precautions. First, and in line with Haken et al.’s (1985) guidelines, we linearly and gradually manipulated TBP in order to examine the dynamical properties of perceived PM. Second, we created high-quality virtual soccer events that psychologically engaged coaches in a way that resembled how coaches could typically experience a real-life soccer match.

## Materials and Methods

### Participants

Forty certified French soccer coaches, who reported having substantial experience in coaching soccer players (only males; *M* = 15.3 years, *SD* = 4.6 years), voluntarily took part in the study (*M*_age_ = 34.20 years, *SD*_age_ = 11.31 years). All coaches reported to possess UEFA coaching licenses (UEFA Pro A or UEFA A) and to participate in national competitions (French League 2 and National levels). They were randomly assigned to the different experimental conditions. For the recruitment of participants, we contacted by phone coaches of several soccer clubs of the north of France and their presidents informed them that we were running a study aiming to examine how coaches could perceive some game scenarios. We also explained that this study would help get a better understanding of coaches’ functioning within important matches. Then, we invited coaches to take part in the study and all of them agreed with the authorization of their presidents.

### Experimental Setup and Design

The study design was approved by the Ethics Committee of the DeVisu laboratory of the University of Valenciennes. The study took place in a university room and participants took part in the experiment individually. The experiment was conducted using a video projection system (Sony VPL-EX120 XGA LCD) that was placed at a distance of 3 m from the wall on which 2 m × 1.6 m images were displayed.

#### Creation of Team Ball Possession Simulations

The TBP simulations were created using PlayStation 4 console with FIFA 18 soccer simulator (Electronic Arts). In order to create a set of 60s-TBP simulations, the experimenters requested the assistance of two e-soccer experts (*M*_age_ *=* 26.8 years, *SD*_age_ *=* 2.5 years) of the FIFA 18 video game who could report playing e-soccer regularly within the week (*M*_practice_ = 10.0 hours, *SD*_practice_ = 1.3 hours) for at least 10 years^1^ (*M* = 12.8 years, *SD* = 1.4 years).

**Figure 2:**
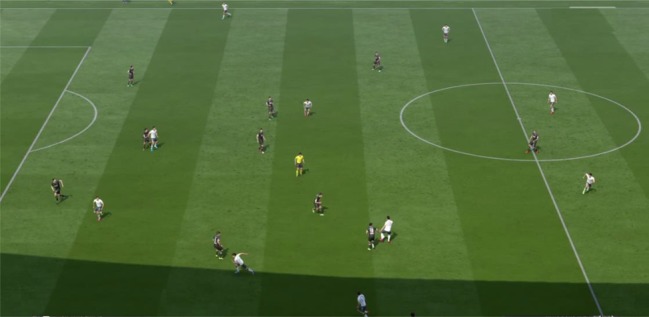
Example of display during the match.

These two experts were considered as technical collaborators of the study. As a result, the experimenters disclosed the objectives of the study to them and emphasized on the importance of not divulgating those objectives to anybody. Then, the experimenters asked the experts to create 60s-TBP simulations involving an opposition between two identical virtual teams that had the same formation (4-4-2) but differentiated based on the color of their equipment (gray vs. white) (see Figure 2). In addition, the two teams included identical virtual players who had no physical resemblances with real players (see Figure 2). This precaution aimed at avoiding any kind of “apophenia” bias (i.e., tendency to perceive links, connections, and meanings between unrelated things) based on the recognition of renowned players, which may lead to any biases consisting in treating ambiguous cues in favor of or against one’s own position. In addition, the experimenters asked the two experts to use either the color white or the color gray (which are achromatic colors) as jersey colors of the two teams. We avoided chromatic colors, such as red, blue, or green, since studies evidenced that such colors could influence affective judgments and physiological states (e.g., Recours and Briki, 2015; Briki and Hue, 2016; Briki and Majed, 2019). Furthermore, the experimenters demanded that TBP simulations would contain only passes, which had to be carried out in a diversity of directions in the region of the center circle of the football pitch (see Figure 2). Maintaining the ball in this region of the pitch while varying the percentage of TBP aimed to avoid making a confusion between a given amount of TBP (e.g., large possession) and a physical movement of the team toward or away from the opponent’s goal, which might be interpreted in terms of level of control over the game. Imagine, for instance, that the team “A” possesses the ball and evolves toward the penalty box of the team “B.” In such a situation, one might perceive that “A” would have a higher control over the game than would “B.” Nonetheless, we could not know if such a perception would be due to the rate of TBP or to the movement of “A” toward the penalty box of “B”; we sought to avoid such a confusion. Lastly, the two e-soccer experts were requested to create seven TBP simulations containing different amounts of TBP [e.g., simulation 1: TBP of 0 s (or of 60 s) for the team “White” (or “Gray”), see Table 1 for all details].

**Table 1:** Characteristics of team ball possession simulations.

	Team ball possession (TBP)	
	Team “White”	Team “Gray”
Simulation 1	0 s (or TBP_0%_)	60 s (or TBP_100%_)
Simulation 2	10 s (or TBP_16.7%_)	50 s (or TBP_83.4%_)
Simulation 3	20 s (or TBP_34.4%_)	40 s (or TBP_66.7%_)
Simulation 4	30 s (or TBP_50%_)	30 s (or TBP_50%_)
Simulation 5	40 s (or TBP_66.7%_)	20 s (or TBP_34.4%_)
Simulation 6	50 s (or TBP_83.4%_)	10 s (or TBP_16.7%_)
Simulation 7	60 s (or TBP_100%_)	0 s (or TBP_0%_)

After a training period that lasted 2 weeks, the e-soccer experts created TBP simulations meeting all the above-mentioned requirements. Afterward, the experimenters modified the contents of the created simulations by using a scenario editing software in order to remove not only the sound, but also any information of score, time, and team names (see Figure 2). Such editing actions aimed at avoiding exposing participants to critical scoring or temporal cues, which are reputed to influence PM experience (e.g., Miller and Weinberg, 1991; Markman and Guenther, 2007). Additionally, such editing aimed at increasing participants’ attention toward TBP.

#### Momentum Sequences

TBP simulations were designed to induce an experience of either a positive PM [with a sequence evolving from 0 s of TBP (or TBP_0%_) to 60 s of TBP (or TBP_100%_)] or a negative PM [with a sequence evolving from 60 s of TBP (or TBP_100%_) to 0 s of TBP (or TBP_0%_)]. The TBP configurations conformed to the methodological requirements of the synergetic paradigm, in such a way that the momentum sequences contained seven linear and gradual thresholds (i.e., TBP_0%_, TBP_16.7%_, TBP_33.4%_, TBP_50%_, TBP_66.7%_, TBP_83.4%_, and TBP_100%_; see Table 1). The momentum scenarios were created in such a way that the two team conditions—the team “white” condition (wearing white) and the team “gray” condition (wearing gray)—could induce experiences of positive PM and negative PM. As a result, we created four potential experimental conditions: (1) gray-positive-PM (*n* = 10), (2) white-positive-PM (*n* = 10), (3) gray-negative-PM (*n* = 10), and (4) white-negative-PM (*n* = 10).

#### Design-Related Precautions

Despite the existence of four potential conditions, we considered Momentum Sequence as the only independent variable of the study. We did not take account of the color of the uniform (white vs. gray) as an independent variable because no theoretical reasons justified the necessity to examine it. However, we examined positive PM and negative PM as a function of both team color perspectives to tackle two methodological issues that went unnoticed in a previous study that used a similar experimental design, notably the study of Gernigon et al. (2010). Firstly, in their study, Gernigon et al. (2010) asked participants to imagine being the table tennis player wearing either red (in the positive momentum sequence condition) or blue (in the negative momentum sequence condition), leading participants to be exposed to different game situations according to the momentum sequence conditions (i.e., positive vs. negative momentum sequences) at identical thresholds. A potential bias concerns the fact that the authors did not neutralize the content of actions from a momentum condition to another one. Said differently, the actions seen by a participant from the perspective of the positive momentum sequence were different from the actions seen by a participant from the perspective of the negative momentum sequence, at a given threshold. Secondly, a specific color was associated with a specific momentum sequence (e.g., the experience of positive PM was always related to the “wearing red” situation), which could not have allowed to differentiate the color effects from the momentum sequence effects. A way to neutralize such potential biasing effects would be, first, to dissociate the links between colors and momentum conditions (i.e., the color X is unrelated to a specific momentum condition) and, second, to analyze data by taking into account only one independent variable, i.e., Momentum Sequence.

### Procedure

Upon his arrival at university, the participant was randomly assigned to one of the four experimental conditions and received basic instructions about the procedure. First, we told him that he would watch a series of game phases involving two teams wearing either white or gray. Second, we asked him to imagine being the coach of the team wearing either white or gray (depending upon the assigned condition) and that his team has reached the final of the most important competition of the history of the club. Third, we told him that he would answer questions about his perceptions and thoughts after every game phase (see Measures section for more details). Then, the participant signed a written informed consent and started the experimental task after which he was thanked for his participation. After the study’s completion, all participants received a personal e-mail in order to present the purposes of the study and its main findings.

### Measures

Perceived PM was measured by the following single item: “In that moment, which team seems to be progressing the most toward victory?” This item was answered to on a 7-point Likert scale ranging from “−3” (“*certainly the opposing team*”) to “+3” (“*certainly my team*”) with a neutral midpoint of “0” (“*neither the opposing team, nor my team*”). The measurement of strategic choice consisted in asking the participant whether he wanted either to maintain (i.e., stick choice) (coded as “1”) or to change (i.e., switch choice) (coded as “2”) the current organization of his team. At the end of the experiment, the participant responded to a manipulation check question that assessed his degree of immersion in the task (Briki et al., 2014b): “To what extent did you succeed in imagining yourself as being the coach you were supposed to be in the final of the most important competition of the history of team?” (“1” = “*not at all*”; “7” = “*very much so*”).

## Analyses

We computed 2 × 7 ANOVA (Momentum Sequence: Positive vs. Negative × TBP: 0–100%) with repeated measures on the “Momentum Sequence” factor for perceived PM and strategic choice (with IBM SPSS Statistics 25). Mauchly’s sphericity tests accompanied the analyses of variance, and we applied Greenhouse-Geisser corrections whenever the assumption of homogeneity of variances was violated. No data were excluded from the analyses.

### Identification of Different Types of Change

*Post hoc* comparisons (Bonferroni’s tests) identified significant changes. A significant difference between two adjacent TBP thresholds would reflect an abrupt change, whereas a significant difference appearing between two distant TBP thresholds would reflect a slight change (e.g., Gernigon et al., 2010). Notably, the observation of slight vs. abrupt changes occurs independently from the observation of linear vs. nonlinear changes. Linearity (or nonlinearity) refers to a proportional (or disproportional) relationship between causes and consequences (e.g., Kelso, 1995; Nowak and Vallacher, 1998). For instance, abrupt or slight changes between TBP thresholds may reflect a linear dynamic as long as the changes are regular and progressive. However, abrupt or slight changes followed or preceded by a period characterized by no changes (i.e., period of stability) would reflect a nonlinear dynamic. In addition to *post hoc* comparisons, trend analyses examined the fit of linear and nonlinear functions (e.g., Gernigon et al., 2010).

### Identification of Dynamical Patterns

Previous studies showed either a negative hysteresis pattern or a critical boundary pattern (e.g., Gernigon et al., 2010; Briki et al., 2013, 2014a).

#### Negative Hysteresis

To support the existence of negative hysteresis, nonlinear changes would occur at different values of TBP according to the positive vs. negative momentum sequences. Mathematically, negative hysteresis means that a value of *x* would be always associated with two values of *y* throughout the momentum sequences, except when *x* is minimal and maximal (see Figure 1A). Negative hysteresis would be characterized by nonlinear changes occurring at the beginning of the two momentum sequences (see Figure 1A). Statistically, the identification of negative hysteresis would require to observe: (1) a significant Momentum Sequence × TBP interaction effect, with significant differences between the momentum sequences around the middle of the sequences only; (2) a significant main effect of Momentum Sequence, with mean scores being higher in the positive momentum sequence than in the negative momentum sequence; and (3) a significant main effect of TBP, with nonlinear changes at the beginning of each sequence.

#### Critical Boundary

To support the existence of critical boundary, nonlinear changes would take place at identical values of TBP for the positive and negative momentum sequences. Mathematically, critical boundary means that a value of *x* would always be associated with a value of *y* throughout the whole momentum sequences (see Figure 1B). As a result, critical boundary would involve a nonlinear change occurring at a specific value of *x* in both momentum sequences (see Figure 1B). Statistically, identifying a pattern of critical boundary would necessitate to observe: (1) a nonsignificant Momentum Sequence × TBP interaction effect; (2) a nonsignificant main effect of Momentum Sequence; and (3) a significant main effect of TBP, accompanied by a nonlinear change.

#### Identification of Asymmetrical Patterns

The observation of asymmetrical patterns would reflect the existence of the property of history-dependence. Studies conducted on PM identified asymmetrical effects with a greater sensitivity to either positive stimuli (e.g., Briki et al., 2014a, 2016; with supporters as participants) or negative stimuli (e.g., Briki et al., 2013; with athletes as participants). Statistically, a change that takes place closer to the beginning than to the end of a sequence would reflect a greater sensitivity of the anticipated future (reflecting a propensity to change), whereas the opposite pattern would indicate a greater sensitivity to the past events (reflecting a resistance to change). Additionally, a change occurring closer to the beginning than to the end of the positive (or negative) momentum sequence would reflect a greater sensitivity to positive (or negative) events. Moreover, the greater the abruptness of the change, the higher the sensitivity of the variable under study to the stimulus (e.g., amount of TBP) at a given time.

## Results

### Manipulation Check

The manipulation check revealed that participants successfully immersed in the experimental task (*M* = 6.42, *SD* = 1.48).

### Did Team Ball Possession Affect Perceived Psychological Momentum and Strategic Choice?

**Figure 3:**
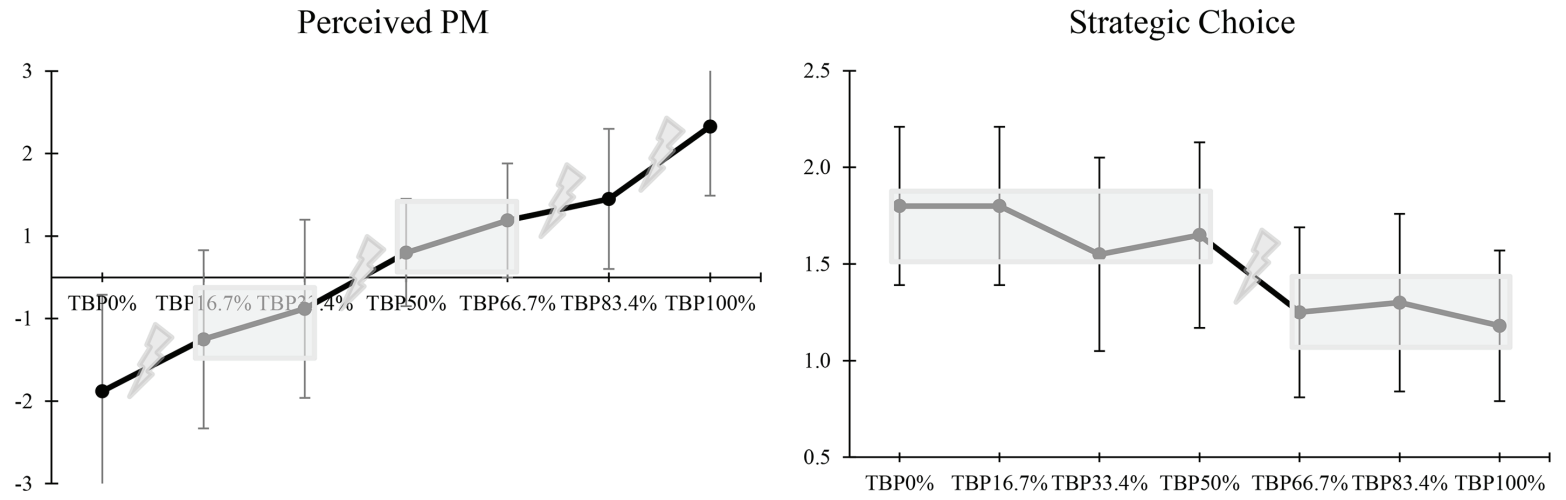
Fluctuations of perceived PM and strategic choice according to team ball possession (TBP). For perceived PM, the used 7-point Likert scale was ranging from “−3” (“*certainly the opposing team*”) to “+3” (“*certainly my team*”) with a neutral midpoint of “0” (“*neither the opposing team, nor my team*”). For strategic choice, the used 2-point Likert scale was ranging from “1” (stick choice) to “2” (switch choice). The positive momentum sequence refers to the evolution of TBP percentage from 0 to 100%, whereas the negative momentum sequence refers to the opposite evolution (100 to 0%). The lightning bolt means the presence of an abrupt change, whereas the rectangle displays the existence of a stationary phase.

ANOVA revealed a significant main effect of TBP for perceived PM (*F*_(3.32, 126.15)_ = 56.47, *p* = 0.000, partial *η^2^* = 0.60) and strategic choice (*F*_(4.46, 126.15)_ = 16.56, *p* = 0.000, partial *η^2^* = 0.30). Specifically, the analyses revealed that perceived PM increased (or decreased) more and more in the positive (or negative) momentum sequence. They also showed that coaches tended more and more to opt for stick (or switch) choices in the positive (or negative) momentum sequence (see Table 2 and Figure 3). Moreover, the main effect of Momentum Sequence (perceived PM: *F*_(1, 38)_ = 1.42, *p* = 0.241, partial *η^2^* = 0.04; strategic choice: *F*_(1, 38)_ = 0.084, *p* = 0.773, partial *η^2^* = 0.00) as well as the interaction effect of Momentum Sequence × TBP (perceived PM: *F*_(3.32, 126.15)_ = 0.813, *p* = 0.500, partial *η^2^* = 0.02; strategic choice: *F*_(4.46, 228)_ = 0.995, *p* = 0.417, partial *η^2^* = 0.03) were nonsignificant for both variables.

**Table 2:** Means and standard errors of the variables under study.

	Perceived PM		Strategic choice	
TBP percentage	M	SD	M	SD
TBP_0%_	−1.38	1.17	1.80	0.41
TBP_16.7%_	−0.75	1.08	1.80	0.41
TBP_34.4%_	−0.38	1.08	1.55	0.50
TBP_50%_	0.30	0.65	1.65	0.48
TBP_66.7%_	0.69	0.69	1.25	0.44
TBP_83.4%_	0.95	0.85	1.30	0.46
TBP_100%_	1.83	0.84	1.18	0.39

### Did Perceived Psychological Momentum and Strategic Choice Display Nonlinear and Asymmetrical Variations as a Function of Team Ball Possession?

Trend analyses revealed that perceived PM significantly varied as a positive linear function of TBP (*p* = 0.000, partial *η^2^* = 0.79), while strategic choice varied as a negative linear function of TBP (*p* = 0.000, partial *η^2^* = 0.62). The Bonferroni’s tests indicated the presence of three abrupt changes for perceived PM: from TBP_0%_ to TBP_16.7%_ (*p* = 0.002), from TBP_50%_ to TBP_66.7%_ (*p* = 0.028), and from TBP_66.7%_ to TBP_100%_ (*ps* ≤ 0.049) (see Figure 3). As for strategic choice, the *post hoc* comparisons showed the existence of two stationary phases, from TBP_0%_ to TBP_50%_ (*p*s ≥ 0.065) and from TBP_66.7%_ to TBP_100%_ (*p*s = 1.000), separated by an abrupt change between TBP_50%_ and TBP_66.7%_ (*p* = 0.021) (see Figure 3).

## Discussion

The present research examined changes in coaches’ perceived PM and strategic choice (stick vs. switch choices) during a simulated soccer match from a synergetic perspective (Haken et al., 1985; Kelso, 1995). In so doing, this research sought to extend studies that examined the dynamical properties of nonlinearity and history-dependence of PM in athletes and supporters (Gernigon et al., 2010; Briki et al., 2013; Den Hartigh et al., 2014) to the exploration of the PM experience of coaches. In this study, we attempted to examine whether and how TBP could affect PM and strategic choice, and whether coaches would either maintain or change the organization of their teams while experiencing positive PM and negative PM. We also sought to see whether the variables under study could display nonlinear and asymmetrical effects.

The present study showed that TBP affected PM perception of coaches. More specifically, the results revealed that perceived PM abruptly changed early and late in both momentum sequences. In the positive momentum sequence, the results showed that: (1) negative PM early reduced (from TBP_0%_ to TBP_16.7%_); (2) positive PM slightly took place (from TBP_16.7%_ to TBP_83.4%_); and (3) positive PM was reinforced at the end of the sequence (from TBP_66.7%_ to TBP_100%_). In the negative momentum sequence, the results indicated that: (1) positive PM early reduced (from TBP_100%_ to TBP_66.7%_); (2) negative PM slightly took place (from TBP_66.7%_ to TBP_16.7%_); and (3) the strength of negative PM increased at the end of the sequence (from TBP_16.7%_ to TBP_0%_). However, more abrupt changes appeared at the end (or at the beginning) of the positive (or negative) momentum sequence (see Figure 3), suggesting the existence of an asymmetrical effect in the sense that the strength of positive PM would be easier to decrease than to increase. This asymmetry supports the view that undergoing a negative event when everything goes right would have a stronger affective effect than experiencing a positive event when everything goes wrong (Briki et al., 2012). More generally, this asymmetry supports the general view that negative events, compared to equivalent positive ones, would have stronger effects (Kahneman and Tversky, 1979; Baumeister et al., 2001). In dynamical terms, one could thus suggest that negative PM, relative to positive PM, represents a stronger attractor (Briki et al., 2013). Moreover, this study supports previous studies that showed that TBP corresponded to a key to success in soccer (e.g., Jones et al., 2004; Travassos et al., 2013). This also supports the view that outcome-related patterns are not the only factors that are responsible for the emergence of the perception that one is making progress toward his or her goal, and thus that PM can be triggered by a wide range of factors (e.g., Taylor and Demick, 1994; Jones and Harwood, 2008; Briki et al., 2012).

In addition, the results indicated a negative relationship between TBP percentage and levels of strategic choice, in the sense that the greater the positive PM (or the negative PM), the lesser (or the greater) the coaches’ tendency to make a change in the organization of their teams. This supports Attali’s (2013) result that revealed that coaches were less likely to make a change when they perceived success. However, our result runs counter to findings of other investigations (Attali, 2013; Guenther and Kokotajlo, 2017) showing that positive PM entailed behavioral changes in actors. However, this difference supports the view that the experience of PM can be sensitive to the type of task involvement (e.g., actor vs. spectator) (Briki et al., 2014a). Moreover, our results showed an abrupt change in strategic choice between TBP_50%_ and TBP_66.7%_ preceded and followed by a stationary phase, thus indicating the existence of a dynamical pattern of critical boundary including an asymmetrical effect. Thus, coaches appeared to be more sensitive to negative events than to equivalent positive events, thereby leading them to show greater tendencies to opt for switch choices. The way coaches appeared to react to positive PM and negative PM is compatible with the tenets of the cumulative prospect theory (Kahneman and Tversky, 1982) that predicts that individuals are less (or more) likely to take risks by making changes when the gains (or the losses) are highly probable.

Finally, this study showed that PM could be induced by game-related events (e.g., TBP) and could powerfully influence coaches’ perceptions and strategic decisions. Nonetheless, this research contains limitations. Its major one is the use of a simulated football match using a virtual platform, which could have reduced the impact of soccer game phases on coaches’ perceptions, thoughts, and feelings, though we collected a self-reported measure of immersion degree within the task. However, the participants of this study reported high mean scores for task immersion, and the virtual nature of this design facilitated experimental control and allowed us to create varied situations that resembled the reality of soccer. Despite these methodological precautions, one should be careful not to generalize the present results to all soccer coaches of the real-world sport matches. Thus, future experimental studies should create more naturalistic settings (by using, for instance, virtual reality) in order to examine more closely the phenomenological experience of PM among soccer coaches. Moreover, future studies should take into consideration personality and examine whether and how personality traits, such as temperament (i.e., general neurobiological sensitivity to appetitive or aversive stimuli) (Elliot and Thrash, 2010), could influence strategic choices in coaches. Indeed, further studies should examine how approach temperament (i.e., strong biological sensitivity to appetitive stimuli) and avoidance temperament (i.e., strong biological sensitivity to aversive stimuli) would influence coaches’ strategic choices while experiencing positive PM and negative PM. To conclude, the investigation of the influence of TBP on perceptions and judgments is an original avenue of research that deserves greater attention from sport and social psychologists. Indeed, it is always surprising to see that some coaches often succeed to make appropriate choices within high-stake competitions when many others fail.

## Author Contributions

BZ collected the data and WB performed the data analysis, interpreted the results, drafted, and revised the manuscript. WB and BZ conceived the study and its design and approved the final version of the manuscript.

### Conflict of Interest Statement

The authors declare that the research was conducted in the absence of any commercial or financial relationships that could be construed as a potential conflict of interest.

## Footnotes

^1^We required 10 years of intense involvement in e-soccer to select our experts since studies estimated that talent and expertise would emerge around 10 years of deliberate practice in a given activity (e.g., Ericsson, 2008).
